# Effets du Parecoxib dans la Prévention des Adhérences abdominales postopératoires: étude expérimentale randomisée chez les rats

**DOI:** 10.11604/pamj.2015.22.180.6481

**Published:** 2015-10-22

**Authors:** Willy Arung, François Tshilombo, Etienne Odimba

**Affiliations:** 1 Département de Chirurgie Générale, Cliniques Universitaires de Lubumbashi, Faculté de Médecine, Université de Lubumbashi, Lubumbashi, République Démocratique du Congo; 2 Centre de Recherche et Développement en Chirurgie (CREDEC), GIGA- Cardiovascular Sciences, Université of Liège (ULg), Liège, Belgium

**Keywords:** Adhérence, postopératoire, complications, prévention, expérimentation chirurgicale, modèle animal, cyclooxygenase-2, antiinflammatoire non stéroïdien, adherence, postoperative, complications, prevention, surgical experimentation, animal model, cyclooxygenase-2, Non-steroidal anti-inflammatory drug

## Abstract

**Introduction:**

Bien d’études ont été menées sur les adhérences intrapéritonéales, mais aucune unanimité n'est encore acquise sur leur prévention. Le but de notre étude a été d’évaluer le potentiel effet d'un antiinflammatoire, parecoxib dans la prévention des adhérences ainsi que sur la cicatrisation chez des rats.

**Méthodes:**

Dans un modèle expérimental d'adhérences postopératoires secondaires à des lésions péritonéales par brûlure, 30 rats furent randomisés en trois groupes suivant le mode d'administration de parecoxib (groupe contrôle; intrapéritonéal; intramusculaire.

**Résultats:**

Le parecoxib a significativement diminué la quantité (p < .05) et la sévérité (p < .01) des adhérences postopératoires dans les deux modèles expérimentaux. Au total, 21 rats ont développé des adhérences, respectivement 9 (100%) dans le groupe A, 5 (50%) dans le groupe B et 7 (70%) dans le groupe C (p = 0.05). Du point de vue de la formation des adhérences au site du traumatisme, dix-neuf rats en ont développé: 9 (100%) dans le groupe A et 5 (50%) pour chacun de deux autres groupes B et C. Une différence significative a été constatée en comparant ces groupes deux à deux: A vs B (p < 0.05); A vs C (p < 0,05). Parecoxib n'a pas compromis la cicatrisation intestinale, ni cutanée.

**Conclusion:**

Cette étude a montré que le parecoxib pouvait réduire la formation des adhérences postopératoires. La confirmation de la sécurité du parecoxib sur les anastomoses intestinales doit être investiguée au cours d'autres expérimentations.

## Introduction

Les adhérences postopératoires sont fréquentes après chirurgie abdominale. Elles ont un impact clinique et économique important. Plusieurs moyens thérapeutiques ont été utilisés pour leur prévention tant dans des modèles animaux que dans des essais cliniques [1–4]. Néanmoins, à ce jour aucune stratégie définitive n'a encore été proposée comme moyen idéal de prévention des adhérences après chirurgie abdominale [5]. Les adhérences postopératoires résultent de la réaction inflammatoire à toute lésion péritonéale. Elles se basent en général sur la diminution de la capacité de l'organisme à accomplir la fibrinolyse. La fibrine constitue donc la protéine ou l’élément capital dans leur formation [5, 6]. En conséquence, plusieurs médicaments dont les glucocorticoïdes et les anti-inflammatoires non stéroïdiens (AINS) ont été évalués comme moyens de prévention de ces adhérences postopératoires. Les résultats de ces études sont en général contradictoires. Néanmoins, certains AINS ont montré une efficacité préventive intéressante [7–9]. Le nimésulide (4-nitro-2-phenoxymethanesulphonanilide), un AINS inhibiteur sélectif de la cyclo-oxygénase-2, a été efficace dans la lutte contre les adhérences chez le rat [10]. Cependant, suite à sa toxicité hépatique, le nimésulide a été retiré dans beaucoup de pays européens et aux USA. Le but de cette étude a été d'investiguer dans un modèle animal, l'efficacité d'un autre inhibiteur sélectif de la cyclo-oxygénase-2, le parecoxib, ou (N-((4-(5-methyl-3-phenylisoxazol-4-yl) phenyl) sulfonyl) propanamide) (Dynastat^®^, Pfizer), dans la prévention des adhérences péritonéales postopératoires.

## Méthodes

L’étude a été menée au Centre de Recherche du Département de Chirurgie (CREDEC) de l'Université de Liège, Belgique, après approbation du protocole par le comité d’éthique animale Institutionnel. La garde des animaux a été conforme aux recommandations internationales sur l'expérimentation animale [11].

**Protocole expérimental:** trente rats mâles adultes Spargue-Dawley ont été répartis de manière aléatoire en trois groupes de dix. Ils ont subi une première chirurgie devant créer une lésion péritonéale dans le but de provoquer des adhérences. Les groupes d’étude ont été formés de la manière suivante: (A) groupe contrôle, aucune mesure préventive appliquée; (B) groupe de rats traités par du parecoxib administré en intrapéritonéal (IP) à la dose de 0,25 mg/kg; (C) parecoxib administré en intramusculaire (IM) à la dose 0,25 mg/kg. Toutes les interventions chirurgicales et l'euthanasie ont été exécutées sous anesthésie générale par injection intrapéritonéale du pentobarbital (Nembutal^®^, 60mg/kg de poids corporel). Ces interventions ont été effectuées dans des conditions strictes d'asepsie. Les animaux ont été placés sur table chauffante avec contrôle de température corporelle par un monitoring rectal et celle-ci a été maintenue entre 36 et 38°C.

**Première chirurgie:** sous anesthésie générale, les rats ont été rasés et fixés sur la table opératoire en décubitus dorsal. Par une laparotomie médiane, le péritoine du flanc droit a été cautérisé au bistouri électrique unipolaire sur un diamètre de 1,5 cm. Dans le groupe B, 100 g (0,25 mg/kg) de parecoxib ont été injectés dans la cavité péritonéale juste après la cautérisation péritonéale, et deux fois par jour pendant les cinq premiers jours postopératoires. Dans le groupe C, 100 g de parecoxib étaient administrés par voie IM dans les suites de la lésion péritonéale (une fois en peropératoire et deux fois par jour, pendant les cinq premiers jours postopératoires). Nonante minutes après la cautérisation, la cavité abdominale a été fermée en deux plans, sans suture péritonéale, avec un fil non résorbable 3-0.

**Seconde laparotomie:** les animaux ont été strictement gardés et surveillés. Au dixième jour, tous les animaux ont subi une seconde intervention sous anesthésie générale. Une laparotomie médiane xypho-pubienne a été pratiquée et la cavité abdominale a été explorée dans son entièreté. Les adhérences péritonéales ont été évaluées et gradées. A la fin de l'expérimentation, les animaux encore sous anesthésie générale ont été euthanasiés par exsanguination (section de l'aorte abdominale).

**Gradation des adhérences (scores):** les adhérences ont été gradées au cours de la seconde laparotomie. La présence ou l'absence d'adhérences a été enregistrée et gradée. Le type, la résistance (évaluant la sévérité des adhérences) et l'extension des adhérences ont été évalués tels que décrits dans le Tableau 1.

**Tableau 1:** Évaluation qualitative et quantitative des adhérences

Catégorie	Description	Score
**Extension**	Aucune adhérence	0
	1 -25% de la surface péritonéale lésée	1
	26 -50% de la surface péritonéale lésée	2
	51-75% de la surface péritonéale lésée	3
	76 -100% de la surface péritonéale lésée	4
**Type**	Aucune adhérence	0
	Adhérence fine et lâche	1
	Adhérence dense	2
	Adhérence vasculaire	3
**Résistance**	Aucune adherence	0
	Facile à séparer	1
	Nécessité de traction	2
	Nécessité de dissection au ciseau	3
**Total**	*Extension + Type + Résistance*	*0 – 10*

**Analyse statistique:** les données sont décrites en nombre absolu, en moyenne ± écart type et en médiane. La médiane et les quartiles sont présentés en graphique (Boxplot). La différence entre groupes a été établie par le test de Khi ou le test Fisher. Des analyses statistiques sur le type, l'extension et la sévérité des adhérences ont été effectuées à partir de l'analyse des variances (ANOVA) ou du test non paramétrique de Kruskal-Wallis (par le logiciel SPSS 19, Inc,Chicago, IL, USA,). Toute valeur de p < 0.05 a été considérée comme Statistiquement significative.

## Résultats

**Evolution postopératoire et taux de mortalité:** un rat du groupe A est décédé au cours de l'induction de l'anesthésie. Cet animal a été exclu de l'analyse des résultats. Les autres animaux ont survécu, sans aucune complication, jusqu'au dixième jour postopératoire.

**Formation des adhérences postopératoires:** au total, 21 rats ont développé des adhérences, respectivement 9 (100%) dans le groupe A, 5 (50%) dans le groupe B et 7 (70%) dans le groupe C (p = 0.05). Il n'y avait pas de différence significative entre les groupes B et C (p > 0.05). Du point de vue de la formation des adhérences au site du traumatisme, dix-neuf rats en ont développé: 9 (100%) dans le groupe A et 5 (50%) pour chacun de deux autres groupes B et C. Une différence significative a été constatée en comparant ces groupes deux à deux: A vs B (p < 0.05); A vs C (p < 0,05). Mais la différence n'a pas été significative entre les groupes B et C (p > 0.05). Quinze rats au total ont développé des adhérences au niveau de la cicatrice médiane de laparotomie, respectivement 8 (89%) dans le groupe A, 3 (30%) dans le groupe B et 4 (40%) dans le groupe C (p < 0.05).

**Figure 1:**
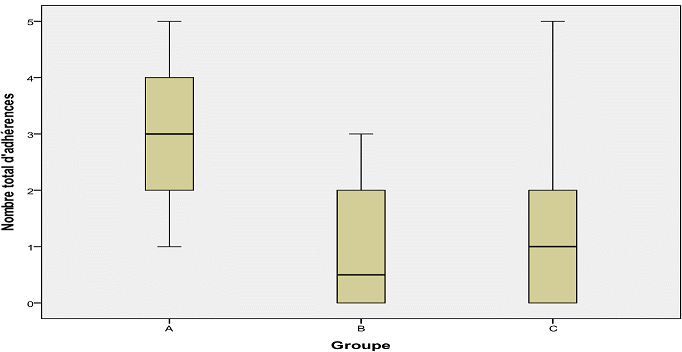
Nombre total des adhérences

**Figure 2:**
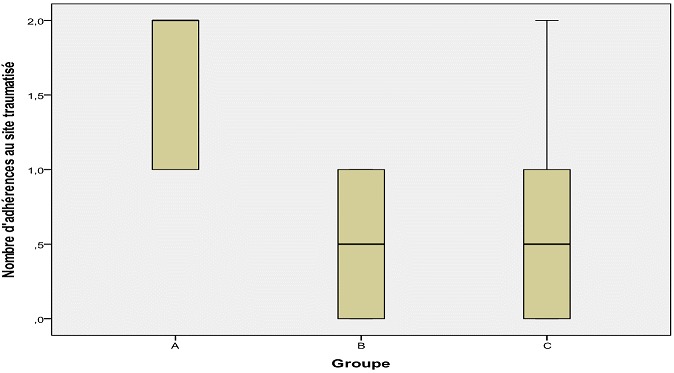
Nombre d'adhérences au site du traumatisme

**Nombre d'adhérences:** au total 56 adhérences ont été formées dans les trois groupes d’étude: 26 dans le groupe A, 12 dans le groupe B et 18 dans le groupe C. Les moyennes des nombres d'adhérences par groupe ont été: 2,89 ± 1,45 groupe A, 0,90 ± 1,10 groupe B et 1,50 ± 1,84 groupe C (p < 0,05). La différence a été également significative entre les groupes A et B (p < 0,01) et A et C (p = 0,05), mais non significative entre B vs C (p > 0,05). Les médianes par groupe sont présentées dans la Figure 1. Au niveau du site traumatisé, 26 adhérences ont été formées: 14 dans le groupe A, 5 dans le groupe B et 6 dans le groupe C. Les moyennes observées dans chaque groupe étaient: groupe A: 1,56 ± 0,52; groupe B: 0,50 ± 0,52 et groupe C: 0,60 ± 0,69 (p = 0,001). Les différences observées entre groupes étaient: A vs B, (p = 0,001); A vs C (p < 0,01) et B vs C (p > 0,05). Les médianes ont aussi été différentes entre groupes (Figure 2).

**Figure 3:**
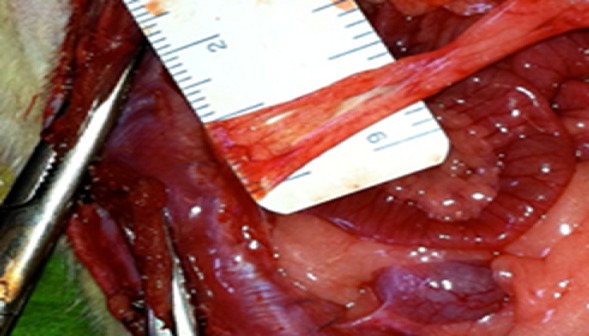
Extension des adhérences

**Figure 4:**
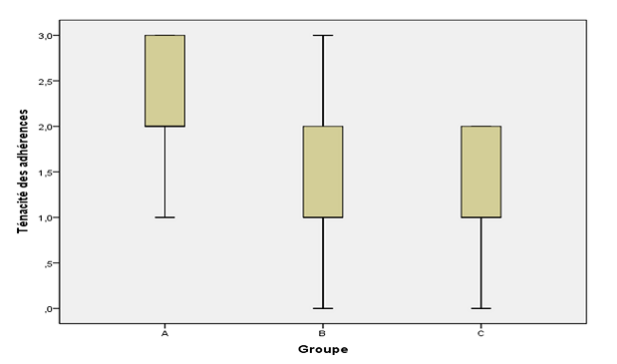
Résistance des adhérences postopératoires

**Figure 5:**
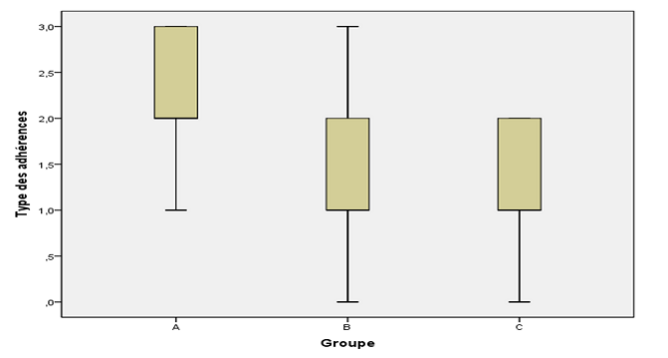
Type des adhérences postopératoires

**Quantification des adhérences:** les scores moyens d'extension des adhérences (Figure 3) ont été: 2,29 ± 1,38 dans le groupe A;1,23 ± 0,83 dans le groupe B et 0,62 ± 0,76 dans le groupe C. La résistance des adhérences a été évaluée de la manière suivante: 2,31 ± 0,67 dans le groupe A; 1,20 ± 0,86 dans le groupe B et 1,25 ± 0,63 dans le groupe C. Les scores moyens d'extension et de résistance des adhérences ont été significativement plus faibles dans les groupes B et C que dans le groupe A (p = 0.001, p < 0.001, respectivement), mais aucune différence significative n'a été observée entre les groupes B et C (p > 0,05). Le score médian de la résistance était de 2 dans le groupe A et de 1 dans les deux autres groupes (Figure 4). Le score médian de type des adhérences est présenté dans la Figure 5. La moyenne par groupe a été mesurée comme suit: 2,31 ± 0,67 dans le groupe A; 1,20 ± 0,86 dans le groupe B et 1,25 ± 0,63 dans le groupe C. Il y avait une différence statistiquement significative entre les groupes A et B (p < 0.001) et entre les groupes A et C (p < 0.001). Aucune différence significative n'a été constatée entre les groupes B et C (p > 0,05).

## Discussion

Les adhérences péritonéales résultent en général d'une réaction inflammatoire à un traumatisme, à une infection, ou à tout corps étranger dans la cavité abdominale. Elles constituent une complication majeure en chirurgie abdominale. Elles sont responsables d'une grande morbidité, suite à leurs multiples complications dont beaucoup se manifestent plusieurs années plus tard [1, 12, 13]. Les adhérences postopératoires représentent un véritable problème en chirurgie digestive. La pathogénie des adhérences a largement été étudiée. Néanmoins, aucune stratégie préventive n'a été définie à ce jour. Divers agents ont été étudiés, des anti-inflammatoires stéroïdiens et non stéroïdiens, des antioxydants, des anticoagulants et des fibrinolytiques [3, 4, 14, 15]. Les résultats obtenus avec ces agents sont contradictoires. Dans cette étude, nous avons constaté l'efficacité du parecoxib, un AINS inhibiteur sélectif de la cyclo-oxygénase-2, dans la prévention des adhérences postopératoires. En effet, sans empêcher complètement la formation des adhérences, l'administration du parecoxib par voie IP ou IM a significativement réduit l'extension et la sévérité des adhérences dans les groupes des rats traités. De plus, 50% d'animaux traités avec le parecoxib n'ont pas développé de manière significative d'adhérences. L'action anti-inflammatoire du parécoxib par son inhibition sélective de la COX-2 contribue certainement aux effets observés. Ainsi, d'autres AINS COX-2 sélectifs ont montré une efficacité dans la prévention des adhérences postopératoires [7, 8, 16]. Guvenal et al. [10] ont observé à ce sujet l'efficacité du nimesulide dans la prévention des adhérences postopératoires. Le nimesulide a réduit la formation des adhérences par une action anti-prostaglandine et par réduction de la production des cytokines “adhésiogéniques”. En outre, il semble que ces AINS COX-2 sélectifs puissent agir tant par voie systémique que par action locale. En effet, Guvenal et al. [10] ont également montré que l'effet préventif de ces AINS était le même quelles que soient les voies d'administration, IP ou IM. Bien que les différences observées du point de vue type, extension et sévérité des adhérences entre les groupes B et C n'aient pas été significatives, les résultats ont paru être meilleurs tantôt pour l'un, tantôt pour l'autre groupe. Le traumatisme provoqué par des injections IP répétées de parecoxib, pendant cinq jours, pourrait expliquer pour certains résultats (extension des adhérences) la supériorité de la voie intramusculaire. Mais comme dit ci-haut, les différences observées entre ces deux groupes B et C n’étaient pas statistiquement significatives. L'efficacité de l'inhibiteur sélectif de COX-2 dans notre modèle expérimental confirme l'importance de la réaction inflammatoire dans la genèse des adhérences postopératoires. De ce fait, le parecoxib peut être indiqué et appliqué dans la prévention des adhérences postopératoires sans effet secondaire particulier, sinon ceux reconnus lors de son usage clinique. Mais Binda et al. [17] n'ont pas trouvé une efficacité des AINS dans la prévention des adhérences, ni spécialement des inhibiteurs sélectifs de COX-2. Cependant, les auteurs tempèrent eux-mêmes leur constat en précisant: “the absence of effect from antiinflammatory drugs was surprising and these data should be interpreted cautiously because results from one species do not necessarily apply to other species”[17].

## Conclusion

Dans ce modèle, le parecoxib a été efficace dans la prévention des adhérences postopératoires. De ces résultats, nous recommandons d'autres études expérimentales et cliniques dans le but de comparer l'efficacité du parecoxib à celle d'autres agents de prévention des adhérences, telles que des barrières mécaniques dont l'efficacité même en clinique a été démontrée. Mais l'avantage notable du parecoxib serrait très certainement son coût faible comparé à celui de certaines barrières dont le “ Sodium hyaluronate/carboxymethylcellulose barrier” (Seprafilm^®^, Genzyme, MA).
